# InvaCost, a public database of the economic costs of biological invasions worldwide

**DOI:** 10.1038/s41597-020-00586-z

**Published:** 2020-09-08

**Authors:** C. Diagne, B. Leroy, R. E. Gozlan, A.-C. Vaissière, C. Assailly, L. Nuninger, D. Roiz, F. Jourdain, I. Jarić, F. Courchamp

**Affiliations:** 1 Université Paris-Saclay, CNRS, AgroParisTech, Ecologie Systématique Evolution, 91405 Orsay, France; 2 Unité Biologie des Organismes et Ecosystèmes Aquatiques (BOREA, UMR 7208), Muséum national d’Histoire naturelle, Sorbonne Université, Université de Caen Normandie, CNRS, IRD, Université des Antilles, Paris, France; 3 ISEM, Univ. Montpellier - CNRS - IRD, Montpellier, France; 4 MIVEGEC, UMR IRD 224 - CNRS 5290 - Univ. Montpellier, Montpellier, France; 5 Santé publique France, Saint-Maurice, France; 6 Biology Centre of the Czech Academy of Sciences, Institute of Hydrobiology, Na Sádkách 702/7, 37005 České Budějovice, Czech Republic; 7 University of South Bohemia, Faculty of Science, Department of Ecosystem Biology, Branišovska 1645/31a, 37005 České Budějovice, Czech Republic

**Keywords:** Environmental economics, Invasive species, Invasive species, Databases

## Abstract

Biological invasions are responsible for tremendous impacts globally, including huge economic losses and management expenditures. Efficiently mitigating this major driver of global change requires the improvement of public awareness and policy regarding its substantial impacts on our socio-ecosystems. One option to contribute to this overall objective is to inform people on the economic costs linked to these impacts; however, until now, a reliable synthesis of invasion costs has never been produced at a global scale. Here, we introduce InvaCost as the most up-to-date, comprehensive, harmonised and robust compilation and description of economic cost estimates associated with biological invasions worldwide. We have developed a systematic, standardised methodology to collect information from peer-reviewed articles and grey literature, while ensuring data validity and method repeatability for further transparent inputs. Our manuscript presents the methodology and tools used to build and populate this living and publicly available database. InvaCost provides an essential basis (2419 cost estimates currently compiled) for worldwide research, management efforts and, ultimately, for data-driven and evidence-based policymaking.

## Abstract (summary)

**Table:** 

Measurement(s)	economic cost of biological invasions
Technology Type(s)	digital curation
Sample Characteristic - Environment	aquatic environment • terrestrial biome • semi-aquatic environment
Sample Characteristic - Location	Earth (planet)

Machine-accessible metadata file describing the reported data: 10.6084/m9.figshare.11627406

## Background & Summary

A biological invasion is the successful introduction, establishment and spread of a species outside its native range, mostly driven by human activity^1^. Invasive species are pervasive drivers of global change, responsible for substantial ecological (*e.g*. biodiversity loss^2^, disturbance of ecosystem functioning^3^), health (*e.g*. spread of diseases^4,5^) and social (*e.g*. declining quality of life^6^) damages almost everywhere in the world. Another important dimension of these impacts is the massive economic losses suffered by our societies (*e.g*. consumption of crops^7^, degradation of infrastructures^8^, decreasing business activities^9^, loss of income^10^). Management efforts aimed at prevention, control and eradication of invaders represent additional, often substantial expenditures for human societies^11–13^. As such, a recent synthesis has shown that invasions of insects alone cost a minimum of US$76.0 billion per year globally^14^.

Despite these enormous impacts, a lack of relevant data and clear public understanding of outcomes associated with invasions provide important barriers to their effective management and mitigation^15^. The need for knowledge and awareness becomes even more crucial in the changing global context in which many more species invasions are expected in the near future^16^. However, despite increasing interest and progress in considering invasions as a crucial problem over the last few decades, the necessity to mobilize more efforts on invasion issues is still urgent^17^. Developing efficient solutions necessarily requires involvement of non-scientists (*i.e*. general public, decision makers, policymakers) worldwide. One option understandable by a wide and varied audience is to describe these impacts in terms of economic costs. Informing people on the potential expenditures and losses dues to impacts of the invaders appears as a fundamental step to *(i)* raise public awareness and compel policymakers to focus a more appropriate attention on invasions, *(ii)* estimate the costs of invasions for specific taxa, geographic regions or activity sectors as well as their drivers, *(iii)* improve assessment of proactive surveillance and control actions as well as prioritising management efforts at relevant scales^18^, and *(iv)* support efficient and cost-effective decision-making^19^.

Consistent, broad-scale approaches and synthetic analyses are increasingly recommended in invasion science to harmonize information, help set priorities of actions and improve coordination of efficient responses at different scales^20,21^. Although significant progress was recently made collating and analysing information on economic data associated with invasions, most of the studies attempting to broadly quantify impacts have so far individually focused on either few model taxa (often at species level)^14^, specific economic sectors^22^ and/or restricted spatial scales (local or regional)^23^. Unfortunately, the even rarer, still widely cited and considered as the only available global estimates^24,25^ are outdated and suffer from methodological flaws that were already highlighted^26,27^. Moreover, whilst being essential for the purpose of policy, management and reporting, open access to full information reported on such type of data remains challenging^28^. To date, such an accessible, current and broad inventory of economic costs associated with invasions exists only for invasive insects^14^.

**Fig. 1:**
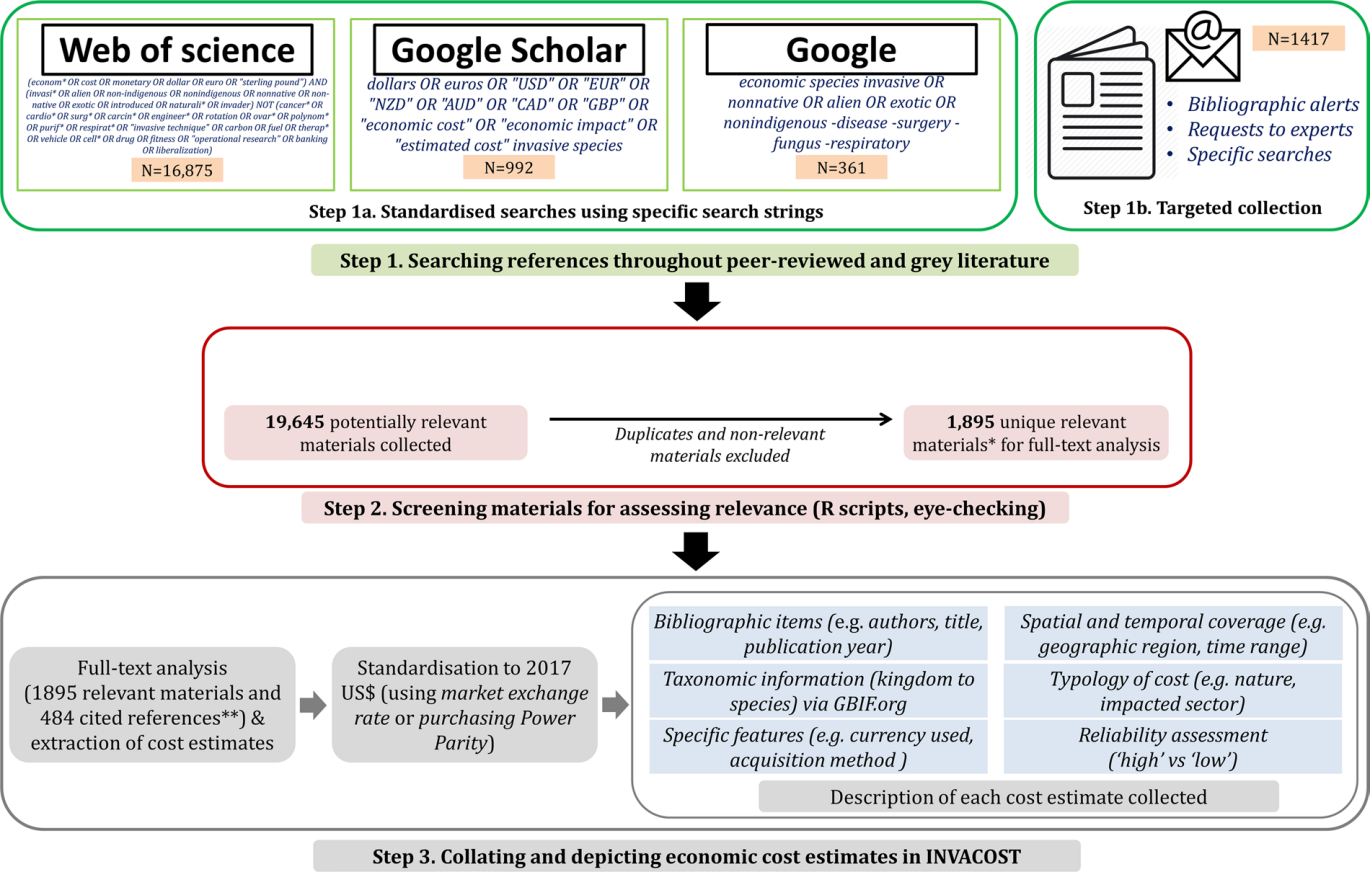
General outline of the different construction steps of InvaCost. * Relevant materials were those *(i)* readable by the review team (*i.e*. written in English or French) for ensuring reliable assessment, *(ii)* containing at least one cost estimate *(iii)* exclusively associated with (at least one) invasive species. This assessment was based on the progressive analysis of titles, abstracts and keywords. Materials whose abstracts were not accessible were conservatively considered as relevant for further full-text analysis. ** Cited references were materials not gathered with our literature search process, but mentioned as original references in the relevant materials (initially collected) we analysed when seeking for cost estimates. Currently, 484 cited references are referenced in InvaCost.

In this paper, we introduce InvaCost as the most up-to-date, comprehensive, harmonised and robust global-scale data compilation and description of economic cost estimates associated with invasive species. InvaCost has been constructed to provide a contemporary and freely available repository of monetary impacts that can be relevant for both research and evidence-based policy making issues. To achieve this goal, we have developed a defensible, systematic, transparent and repeatable collaborative work strategy (Fig. 1). A large pool of both scientific peer-reviewed articles and grey literature (*i.e*. the diverse and heterogeneous body of material available outside, and not subject to, traditional academic peer-review processes^29^) was collected and scrutinized (Table 1). We extracted therein explicit estimations of costs and expenditures associated with invasive species, and then coupled them with a range of descriptors presented in this paper (Online-only Table 1). Here, we provide a full description of the process of InvaCost development, as well as specific details of all materials analysed. This unique, globally representative database (n = 2419 cost estimates currently described) is freely accessible online^30^ and will be regularly updated with contributions from both authors and future users.

**Table 1:** Quantitative summary of the literature search process.

Bibliographic repositories	N	n	Spreadsheets in InvaCost_references*
Web of Science	16.875	1.333	*WoS-collected_refs; WoS-screened_refs*
Google Scholar	992	310	*GS-collected_refs; GS-screened_refs*
Google search engine	361	119	*Go-collected_refs; Go-screened_refs*
Targeted collection	1.917	634	*TC-collected_refs; TC-screened_refs; Cited references*

Search was performed using four bibliographic repositories. N: the number of references initially collected after applying specific search strings (Web of Science, Google Scholar, Google search engine) or as the result of targeted collection. n: the number of relevant materials (*i.e*. materials expected to contain cost estimates based on the contents of titles, abstracts and keywords) derived from N. The figures for the targeted collection include 484 references gathered within the analysed relevant materials. *The spreadsheets in which all details of associated references were recorded^30^ are also referenced.

## Methods

### General scheme

We reviewed the literature published until April 2018 on the economic impacts of invasive species. For reasons of feasibility (linguistic skills of the review team, restriction to a reasonable scale of the review), we conducted all searches in the English language assuming that a large body of knowledge (mostly from international peer-reviewed papers and reports) is written in English. The dates of each search process were systematically recorded. We used the following strategy for all repositories (Fig. 1), while also taking into consideration the specificity of their algorithms.

First, a literature search was performed using three online bibliographic sources successively to minimize the risk of omitting relevant materials (Fig. 1, step 1a): ISI Web of Science platform (https://webofknowledge.com/), Google Scholar database (https://scholar.google.com/) and the Google search engine (https://www.google.com/). We carefully composed appropriate search strings that were consensually retained as the most efficient among a set of potential candidates. A decision was taken following preliminary tests based on a handful of relevant articles provided by consulted subject experts on some taxonomic groups (amphibians, reptiles, fishes and ants). Final selection of search strings comprised those considered to have the largest potential to identify key references. Each search string was set to include a combination of two search terms, related to ‘invasive’ and ‘economics’. For both terms, we used a range of synonyms or related words. For example, for ‘invasive’ we used invasi*, *invader* or *exotic*; for ‘economics’, we used econom*, *cost* or *monetary*. In addition, the search string included exclusion terms to omit mismatches, for example, with studies from the field of medicine that are focused on pathologies or procedures that can be ‘invasive’ for patients. We complemented this search with documents gathered opportunistically (Fig. 1, step 1b). The potentially relevant materials derived from all these sources were combined in a single file and screened for duplicates. Second, retrieved documents were individually assessed at progressive levels (titles, then abstracts, keywords, and finally full text when abstracts were missing; Fig. 1, step 2) based on three criteria. Hence, materials were deemed relevant if *(i)* they matched with the linguistic competencies of the review team (*i.e*. written in English, or French where English language was restricted only to the title and/or abstract) for allowing reliable assessment, *(ii)* they contained at least one cost estimate (studies exclusively providing benefit estimates from direct use or exploitation of invasive species were excluded), and *(iii)* that this cost estimate is exclusively associated with invasive species (estimates merging non-invasive and invasive species, without the possibility of distinguishing the respective contribution of each group to the overall cost, were excluded). To ensure transparency and validity, each document was checked by two reviewers and in case of a disagreement between assessors, a third reviewer was involved. However, it was often difficult to judge from the topic whether the content of an article was relevant and so consequently many more articles were conservatively kept when final agreement was lacking among assessors.

Finally, relevant materials were scrutinized for data on economic costs (Table 1; Fig. 1, step 3). During this step, additional relevant materials were found as cited by the analysed materials. Obtained cost data were collated in a database and the costs were converted to a common and up to date currency (2017 US$), and then depicted by different descriptors. Categories extracted from relevant materials allow search of the database and data pre-selection to facilitating analysis of costs based on taxonomic groups, geographical areas, impacted sectors, types of costs, or other categories. The reliability of cost estimates and all associated information recorded in the final InvaCost database was systematically checked at least twice, and every ambiguous element was discussed to reach a consensus. We also checked all entries in the database to ensure that there were no obvious duplicate reports (i.e. multiple documents reporting the same cost estimate) or mistakes.

Hereafter, we specifically describe each of the steps made to generate InvaCost.

### Literature search

#### Web of Science

We used the Web of Science (hereafter called WoS) to conduct a search for potentially relevant materials on 7 December 2017 (Fig. 1, step 1a). We applied the following search string: (econom* OR cost OR monetary OR dollar OR euro OR “sterling pound”) AND (invasi* OR alien OR non-indigenous OR nonindigenous OR nonnative OR non-native OR exotic OR introduced OR naturali* OR invader) NOT (cancer* OR cardio* OR surg* OR carcin* OR engineer* OR rotation OR ovar* OR polynom* OR purif* OR respirat* OR “invasive technique” OR carbon OR fuel OR therap* OR vehicle OR cell* OR drug OR fitness OR “operational research” OR banking OR liberalization). The terms were searched in the field code “Topic” which includes title, abstract and keywords, and which also comprises ‘Keywords Plus’ that are generated by WoS through an automatic computer algorithm, based on words and phrases that appear frequently in the titles of article’s bibliographic references and not necessarily in the main text of the article itself. To limit the search to relevant fields of research, we used the function ‘refine’ to exclude subject areas not related to economics and/or invasion biology.

We exported all records (n = 16,875) into an Excel worksheet^30^ (Table 1) to identify the relevant materials by a two-step procedure. First, we excluded the references identified only based on ‘Keywords Plus’, which were shown to be poor specific descriptors of the content of articles^31^. We also excluded references identified based on the presence of only a single search term in the topic, as we assumed that words related to both search terms (‘invasive’ and ‘economics’) should be mentioned at least once in the title, abstract and/or keywords of a relevant material. To identify these irrelevant materials within the references collected, we developed a script (see Code Availability) in the R programming language (R v.3.4.3)^32^. Subsequently, 10,592 references were kept for the next screening step based on the described criteria.

In the second step, the topic of every reference selected was checked manually to ensure potential relevance of its contents. This allowed the elimination of documents incorrectly identified as relevant, such as studies without a true monetary assessment, or those focusing on economic estimates not directly attributable to invasive species only. Finally, 1,333 documents were judged as relevant materials (Table 1) and moved to the final data collation step.

#### Google scholar

The Google Scholar database is a large source of grey as well as peer-reviewed literature. Nevertheless, we had to modify our approach in order to address inherent limitations of this database as a search tool (see Haddaway *et al*.^33^ for a comprehensive analysis). Typically, Google Scholar allows limited Boolean operators (no nesting using parentheses permitted) and search strings are limited to 256 characters. Additionally, only the first 1,000 search results can be viewed and the order in which results are returned is not disclosed. We also wanted to maximize novel information by avoiding too much overlap between the references collected with WoS and those gathered here.

In light of the above, we adapted our search string to generate the most efficient outcome, *i.e*. sufficiently pertinent to bring the most relevant items to the top of the result list while not unnecessarily large so as to limit the host of non-viewable results. Thus, the following search string was applied on 26 April 2018, using the advanced search facility to search for selected words anywhere in the article (see https://scholar.google.se/intl/en/scholar/help.html#searching for further details): ***dollars OR euros OR “USD” OR “EUR” OR “NZD” OR “AUD” OR “CAD” OR “GBP” OR “economic cost” OR “economic impact” OR “estimated cost” invasive species***. We specified currencies for prioritising materials with monetary data in the top of the resulting list. These currencies were chosen as they were the most often used to express economic costs in the literature collected from the WoS. Nevertheless, any reference evoking economic costs in other currencies was expected to be also captured by some specific combinations of ‘*economic*’ terms in our search string that we would expect to be mentioned at least once in the full-text of relevant papers. In addition, we included the concomitant presence of ‘invasive’ and ‘species’ terms to restrict the outcomes to papers within the scope of our synthesis. Subsequently, we collected all viewable results (100 pages, n = 992 references of the 668,000 generated), thus going beyond the traditional and arbitrary sample size of first 50–100 results, which is frequently selected in many systematic reviews. We used a web-scraping programme (https://www.webscraper.io/) to extract all the titles’ references returned by the search in an Excel spreadsheet. Because we could not efficiently export the abstract for every reference, we screened them online to assess their potential relevance.

As a result of a search and relevance assessment within Google Scholar, the references, abstracts and specific bibliographic details of 432 documents were added to the sample for further analysis. After excluding duplicates with WoS retrieved references, 310 additional documents were included in the sample as potentially relevant materials (Table 1).

#### Google

We used the Google search engine to complete the standardised literature search. As when searching with Google Scholar, we took into account specific constraints related to the use of this search engine. Moreover, browsing through Google search results can be overwhelming due to the vast amount of information of highly variable quality. We attempted to implement a search strategy that could allow overcoming these limitations as much as possible. We used the following search string: ***economic species invasive OR nonnative OR alien OR exotic OR nonindigenous -disease -surgery -fungus -respiratory***. We added four exclusion terms (*disease, surgery, fungus, respiratory*) identified during preliminary tests to restrict the number of irrelevant studies, associated with medical research. We did not use a range of economics-related terms, such as impact or cost, as they returned overly large numbers of mismatches.

The web search was conducted on 8 May 2018 by searching for specified terms within page titles of each document, in order to maximize the likelihood of identifying grey literature. We especially targeted grey literature because searches by the other two platforms mainly led to peer-reviewed publications. We assumed that documents published online by various governmental and non-governmental organisations (NGO), research centres and academic institutes are more likely to contain relevant data than other types of documents such as blogs and catalogues^29^. Therefore, we restricted our search to the documents located on governmental, academic and NGO webpages to ensure that explicit, traceable and expertise-based information was retrieved. We conducted independent searches for each type of webpage by specifying the type of web extension in the advanced search facility (*.gov* for governmental,.*edu* for academic, and.*org* for organisational webpages).

361 search hits were collected (document name, publishing year and URL of the main website homepage, if available) and stored in the database with the same host of dedicated information (Table 1). If the item analysed was a website homepage, we conducted on-line searches of potentially relevant materials within the website database(s), by filters if available, or by using the search bar with combinations of keywords. Websites that did not contain a database or search bar were searched manually. We then eliminated all duplicates resulting from references being listed on multiple websites, or due to typographical mistakes and/or incomplete records when reporting a reference within different repositories. A total of 119 potentially relevant materials was finally obtained (Table 1).

#### Targeted collection

Finally, we sourced other potentially relevant materials that did not originate from the above-described processes (Fig. 1, step 1b). On one side, we dedicated specific efforts on gathering cost estimates for particular taxa or areas for which data previously obtained seemed scarce. First, we made sure that some key species were adequately covered; for example, costs associated with invasive mosquito species responsible for much of the burden of mosquito-borne viral diseases worldwide (*Aedes aegypti* that mainly invaded the intertropical zone from the 15th-17th centuries, and *Aedes albopictus* for which the global dissemination was more recent^34^) were searched in a specific way using WoS and PubMed (https://www.ncbi.nlm.nih.gov/pubmed/) repositories (see supplementary file 1 for details on search strings and matching with PRISMA statements). Second, materials were also retrieved following requests to specialists (*e.g*. Aliens mailing list, https://list.auckland.ac.nz/sympa/info/aliens-l) to bridge gaps identified for Russia and China, two of the five largest countries for which available on-line data were particularly scarce. A typical message first summarized the objectives of our research project and second, requested recipients to provide relevant material and/or suggest further contacts in this regard. On the other side, we also compiled additional materials when establishing the methodology for the project (*e.g*. when testing different search string combinations at initial stages of the work), from the bibliographic alerts set up by the review team. All 1417 documents obtained from this process were entered in the database, with information on the person providing the document (Table 1;^30^). Subsequently, 150 documents identified as not previously retrieved were considered relevant for further, full-text screening (Table 1).

### Extraction of cost estimates

The Online-only Table 1 comprises all the information of InvaCost that we mention further in this article, using simple quotation marks for ‘Columns’ of the database and italic letters for the different categories within each column. The full-text of each relevant material was scrutinized for any cost estimate that could be incorporated into InvaCost^30^. The final stage of inclusion/exclusion took place during this data extraction. When the screened documents reported cost estimates by citing sources that were not retrieved by our literature search, whenever possible we assessed the original sources of data in order to better characterize the reported cost. These novel information sources not initially captured by our literature search were then added to the collection list (Table 1). In such cases, we provided information on all documents that were consulted to trace back the original source (‘Previous materials’). In contrast, if no original cost data were found in the cited source, the document was discarded. For all reported costs where the original source was not available or accessible, we emphasized this in a dedicated column (‘Availability’).

Then, we first extracted raw cost data, *i.e*. how they appear in the material in local currency (‘Raw cost estimate local currency’). When multiple cost estimates were provided for a single instance, we calculated median values (e.g. different cost estimates according to several management scenarios dedicated to the same invasive population) and collated the minimum and maximum estimates provided (columns ‘Min/Max raw cost estimate local currency’). When costs were estimated at different time and/or spatial scales in the same material, we opted to choose – when possible – those estimate(s) that summarise(s) as effectively as possible the figure(s) shown in the study. If such an estimate was not obvious to identify throughout the full-text, we extracted every relevant cost estimate. In these latter cases where several cost estimates were provided in a single study, we also collated the minimum and maximum estimates provided.

Temporal information on the costs were also retrieved: the ‘Period of estimation’ as stated in the material and hence, when possible, the ‘Probable starting/ending year’ of the period of estimation and the ‘Time range’ (*year* if the estimate is given yearly or for a period up to one year, *period* if the estimate is given for a period exceeding a year). The ‘Occurrence’ column gives the status of the cost estimate as *potentially ongoing* (if the cost can be expected to continue beyond the period of estimation) or *one-time* (if the cost was deemed as unlikely to continue). For cost estimates provided without a clear indication on the timeframe considered, or covering periods shorter than a year, we considered them with a *year* ‘Time Range’ and a *one-time* ‘Occurrence’ to avoid the risk of overestimating the duration of collated costs. The ‘Raw cost estimate’– with complementary information on the ‘Time range’, ‘Period of estimation’ and ‘Occurrence’ – can be used to estimate total costs over a given period of time. We then transformed the raw cost estimates to cost estimates per year (‘Cost estimate per year’) by dividing the raw costs with a *period* ‘Time Range’ by the duration of the ‘Period of estimation’ (obtained from the difference between the ‘Probable ending year’ and ‘Probable starting year’). The raw costs with a year ‘Time Range’ were reported as they are, because they are already considered at the scale of a year.

### Description of cost estimates in InvaCost

Each of the cost estimates recorded was characterized by a number of information, including ***(a)*** the reference from which the cost was extracted, ***(b)*** the taxonomy of the associated species, ***(c)*** the spatial and temporal coverage of the study, ***(d)*** the typology of each cost estimate and ***(e)*** the evaluation of the reliability of the estimation method(s). For most of the variables considered in InvaCost, a non-negligible part of the cost estimates was not attributable to a single existing category due to the lack of precise information provided by the authors or because they simultaneously belong to multiple categories. In such cases, we respectively reported them as either *Diverse/Unspecified* or as slash-separated lists of categories (*e.g. Artiodactyla/Carnivora* for the ‘Order’).

Details about the nature of the information retrieved as well as the choices made to characterize each cost are synthesized in Online-only Table 1:

***(a)*** We provided bibliographic information on each reference (*e.g*. ‘Reference title’, ‘Authors’, ‘Publication year’). Others specific details (*e.g*. abstract, journal, download link) are given in a dedicated file^30^ with which the columns ‘Repository’ and ‘Reference ID’ of InvaCost allow correspondence of information.

***(b)*** We normalised and harmonised all taxonomic information on the invasive species (‘Kingdom’ to ‘Species’ level) using the GBIF.org Backbone Taxonomy^35^. At this stage, spelling and other taxonomic errors were corrected. While each cost extracted was generally associated with a single invasive alien species, in some cases the data was related to multiple species without the possibility of disentangling species-specific costs. In this case, we mentioned either all species concerned if explicitly indicated by the author(s), or *Diverse/Unspecified* if not.

***(c)*** We dedicated seven columns to describing the impacted area according to its environment (terrestrial and/or aquatic habitats), the temporal extent as mentioned earlier (*e.g*. ‘Period of estimation’, ‘Time range’) and the spatial coverage from the ‘Geographic region’ (*e.g*., Central America, South America, Oceania-Pacific Islands) - rather than the official continent for better accuracy - down to the exact site (‘Location’) when possible. Each area was related to its country of attachment, leading to some mismatches between the ‘Geographic region’ and ‘Official country’ columns due to the existence of countries with non-contiguous overseas territories. For instance, costs found from invaders in La Réunion (a French oversea department) were attributed to *Africa* as ‘Geographic region’ and France as ‘Country’, while France obviously belongs to European continent.

***(d)*** We characterised the typology of each cost mainly based on the following descriptors. The ‘Implementation’ at the moment of the cost evaluation states whether the reported cost was *observed* (*i.e*. cost actually incurred by an invasive species within its invasive distribution area) *or potential* (*i.e*. not incurred but expected cost for an invasive species beyond its actual distribution area and/or predicted over time within or beyond its actual distribution area). The ‘Acquisition method’ provides information on how the cost data was obtained, *i.e. report/estimation* directly obtained or derived (using inference methods) from field-based information, or *extrapolation* relying on computational modelling. The ‘Impacted sectors’ indicates which activity, societal or market sectors were related to the cost estimate (see Table 2 for details). The ‘Type of cost’ ranges from the economic *damages* and *losses* incurred by an invasion (*e.g*. value of crop losses, damage repair) to different levels of means dedicated to the management of biological invaders (*e.g*. control, eradication, prevention).

**Table 2:** The different market and/or activity sectors mentioned in InvaCost.

Sector	Description
Agriculture	Considered at its broadest sense, food and other useful products produced by human activities through using natural and/plant resources from their ecosystems (*e.g*. crop growing, livestock breeding, beekeeping, land management)
Authorities-Stakeholders	Governmental services and/or official organisations (*e.g*. conservation agencies, forest services, associations) that allocate efforts for the management *sensu lato* of biological invasions (*e.g*. control programs, eradication campaigns, research funding)
Environment	Impacts on natural resources, ecological processes and/or ecosystem services that have been valued by authors such as disruption of native habitats or degradation of local habitats
Fishery	Fish-based activities and services such as fishing and aquaculture
Forestry	Forest-based activities and services such as timber production/industries and private forests
Health	Every item directly or indirectly related to the sanitary state of people such as vector control, medical care and other derived damage on human productivity
Public and social welfare	Activities, goods or services contributing - directly or indirectly - to the human well-being and safety in our societies, including local infrastructures (*e.g*. electric system), quality of life (*e.g*. income, recreational activities*)*, personal goods (*e.g*. private properties, lands), public services (*e.g*. transports, water regulation), and market activities (*e.g*. tourism, trade)

Note that most of the cost estimates recorded in the database are associated with more than a single sector and are thus reported as slash-separated lists of sectors.

**Fig. 2:**
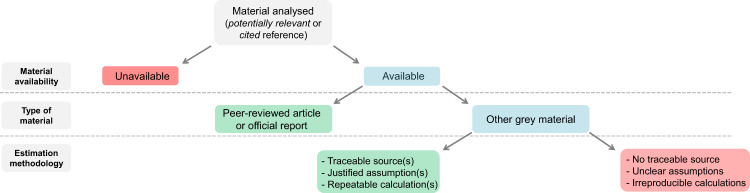
Decision tree approach for assessing the reliability of the method used for estimating each cost. The colour of the boxes indicates which decision was taken: *green* when material was deemed as of *high* reliability, *red* when material was deemed as of *low* reliability, *blue* when taking any decision needs further investigation. The intended purpose of this process was not to evaluate the quality, relevance or realism of the studies performed for providing cost estimates, but rather to assess if the methodology *(i)* has been reviewed and validated by peers or experts prior any publication, or *(ii)* if not, whether this methodology was clearly stated and demonstrated.

***(e)*** Lastly, we evaluated the level of ‘Reliability’ of the methodology reported by the authors to provide cost estimates (Fig. 2). Prejudging the relevance of each cost estimate is not straightforward and could suffer from a high level of subjectivity. Here, we rather aimed to evaluate in the most objective manner whether the approach used for cost estimation was documented and traceable. Hence, materials that could not be accessed for full-text investigation were conservatively considered as of *low* reliability. Alternatively, each cost estimate recorded from any accessible material was qualitatively assessed as of *high* or *low* reliability following a procedure depending on the ‘Type of material’ analysed (*peer-reviewed article* or *grey material*; Fig. 2). Peer-reviewed articles and official documents (*e.g*. institutional or governmental reports) are likely validated by experts before publication. We assumed therefore that all cost estimates collected from these materials may likely be of *high* reliability. Conversely, for grey materials other than official reports, the attribution to one or other of these categories (high vs low reliability) was based on specific analysis of each cost estimate. We checked whether the method estimation was fully described, independently of its comprehensiveness, *i.e*. if the original sources or potential assumptions were properly documented or justified, and/or the calculation methodology was explicitly demonstrated. Here, we opted for a conservative strategy that might be not optimal, as depending mostly on the nature of the publication.

Beyond the factual elements included in the descriptors from *(a)* to *(c)*, those presented in *(d)* and *(e)* (to which we can add the descriptor ‘Spatial scale’) are the result of a conceptual and analytical framework created based on our own experience. This experience was gained when collecting and getting acquainted with the diversity and complexity of situations one can find behind the “economic costs” linked to biological invasions, as well as the strategies used for estimating them. We think that the different subcategories identified therein (*e.g. observed* vs *potential* costs within the descriptor ‘Implementation’) should not be aggregated to limit potential confusions in future analysis. Also, we acknowledge that the possible sub-categories of these descriptors might be improved and adapted according to the scope of future analyses made using InvaCost. We are convinced that the descriptors thus defined and categorised may strongly help in this perspective.

### Standardisation of cost data

Using definitions, data and indicators provided by the World Bank Open Data and the Organisation for Economic Cooperation and Development (OECD), we expressed all retrieved costs (raw costs and costs per year) in US dollars (US$) for the year 2017^30^ using a multi-step procedure. We provided here two ways for standardising cost estimates according to the conversion factor: one based on the market exchange rate (local currency unit per US$, calculated as an annual average), and another based on the Purchasing Power Parity (PPP, local currency unit per US$, calculated as an annual average) that is the rate of currency conversion that standardises the purchasing power of different currencies by eliminating the differences in price levels between countries. Opting for one strategy or the other for further investigation or discussion is beyond the scope of this paper and will befall on the author(s) of future analyses made using InvaCost.

We first converted the cost estimates from local currencies to US$, by dividing the cost estimate with the official market exchange rate (https://data.worldbank.org/indicator/PA.NUS.FCRF?end=2017&start=1960) corresponding to the year of the cost estimation (‘Applicable year’, that is the year of the ‘Currency’ value, but not necessarily the year of the cost occurrence). The cost obtained in US$ of that year was then converted in 2017 US$ using an inflation factor that takes into account the evolution of the value of the US$ since the year of cost estimation. The inflation factor was computed by dividing the Consumer Price Index (CPI, which is a measure of the average change over time in the prices paid by consumers for a market basket of consumer goods and services; https://data.worldbank.org/indicator/FP.CPI.TOTL?end=2017&start=1960) of 2017 by the CPI of the year of the cost estimation.

As an alternative, we also converted costs to 2017 US$ value based on PPP instead of the classical market exchange rates in the initial conversion step. PPP values were primarily collected from data provided by the World Bank (https://data.worldbank.org/indicator/PA.NUS.PPP?end=2017&start=1990), or by the OECD (https://data.oecd.org/conversion/purchasing-power-parities-ppp.htm) when information was not retrievable through the World Bank database. For this purpose, we had to deal with published costs that were expressed in currency that was different from the country where the costs were estimated (*e.g*. published cost in African countries expressed in US or Canadian $). Thus, prior to using PPP as a conversion index, we had to perform a retro-conversion by multiplying the original cost estimate by the official market exchange rate (local currency unit per currency unit used). For PPP-based standardisation, it was not possible to perform the process for all cost estimates as PPP data do not exist for all countries and/or specific periods (we mentioned NA in the database when such information was missing).

In summary, we used the following formula to convert and standardise each cost estimate:

$${C}_{e}=\left({{\boldsymbol{M}}}_{{\boldsymbol{V}}}/{{\boldsymbol{C}}}_{{\boldsymbol{F}}}\right)\,\times \,{{\boldsymbol{I}}}_{{\boldsymbol{F}}}$$

with ***Ce*** = Converted cost estimate (to 2017 US dollars based on exchange rate or Purchase Power Parity), ***M***_***V***_ = Cost estimate (either the ‘Raw cost estimate local currency’ extracted from analysed paper or the ‘Cost per year local currency’ transformed by us), ***C***_***F***_ = Conversion factor (either the official market exchange rate or the purchasing power parity, in US dollars), ***I***_***F***_ = Inflation factor since the year of cost estimation, calculated as CPI_2017_/CPIy with CPI corresponding to the Consumer Price Index and y corresponding to the year of the cost estimation (‘Applicable year’).

We thus provided four columns with the raw cost estimates or the cost estimates per year, expressed in 2017 USD based on the exchange rate or PPP.

### Data summary

InvaCost currently contains 2419 cost estimates (1215 from peer-reviewed articles, 1204 from grey materials), collected from 849 references, of which 1769 estimates were deemed as of *high* reliability. In total, twenty currencies are reported in our database, the majority being US dollars, n = 1348 cost estimates. Not all cost estimates were successfully converted to 2017 US$ as *(i)* conversion data from official sources are available only since 1960 (cost estimates range from 1945 to 2017 in InvaCost) or simply not found for some years and countries, and/or *(ii)* cost data are sometimes simultaneously associated with several countries, constraining the PPP-based standardisations. Hence, respectively 2416 and 2126 estimates were successfully converted using market exchange rates and PPPs. Cost estimates are either direct reports/estimations (n = 2127) or values gathered from extrapolative computations (n = 292). At a taxonomic level, these estimates are associated with 343 species belonging to six kingdoms (Animalia, Bacteria, Chromista, Fungi, Plantae, Riboviria). InvaCost has global coverage (90 countries) and includes continental, insular and overseas territories. Data are associated with terrestrial as well as aquatic (freshwater, brackish and marine) environments. Costs were estimated at different spatial scales (*continental* (n = 35), *country* (n = 1111), *global* (n = 17), *intercontinental* (n = 9), *regional* (n = 67), *site* (n = 836), *unit* (n = 329)). The Table 3 summarises quantitative data and information reported in InvaCost for each geographic region considered (see also Supplementary file 2).

**Table 3:** Quantitative summary of information recorded in InvaCost according to the ‘Geographic region’ of the cost estimates.

Geographic regions	Number of materials	Number of cost estimates	Number of high reliability estimates	Number of taxonomic units
Africa	46	182	163	51
Asia	75	132	111	20
Central America	29	61	51	9
Europe	102	338	255	89
Mixed	33	38	30	19
North America	316	716	438	139
Oceania-Pacific islands	272	867	646	129
South America	45	85	75	16

Central America includes cost data from the Carribean area. ‘Mixed’ contains data concomitantly associated with two or more geographic regions. Taxonomic units refers either to a single species or, if any, to a unique group of species for which specific contribution to the whole cost is not possible to disentangle.

### Possible applications

InvaCost is expected to help bridge the gap between a growing scientific understanding of invasion impacts and still inadequate management actions. This work is thus in line with the aims of a panel of decisions recently adopted by the Convention on Biological Diversity (Decision XIII/13, https://www.cbd.int/doc/decisions/cop-13/cop-13-dec-13-en.pdf) advocating the incorporation of invasion science knowledge into management planning. In addition to offer unique opportunities for future research, InvaCost will provide a strong quantitative and evidence-based support for impacts of invasive species reported in other databases such as the Global Register of Introduced and Invasive Species (GRIIS)^20^, helping refine information in this database. Also, invasive populations recorded in InvaCost but data deficient in the GRIIS should be ultimately classified in that database.

Additionally, InvaCost could be considered as another data-based component, adding novel and significant information on invader impacts categorised by the Socio-Economic Impact Classification of Alien Taxa (SEICAT)^36^. The latter is a classification system, applicable across a broad range of taxa and spatial scales, providing a consistent procedure for translating the broad range of measures and types of impacts into ranked levels of socio-economic impacts, assigning alien taxa on the basis of the best available evidence of their documented deleterious impacts. Quantitative support provided by InvaCost will strongly contribute to impact classification. Ultimately, integrating data from these diverse sources could allow a complete description of the overall impacts of biological invasions at regional and global scales.

### Caveats and directions for further database improvement

Rather than claiming exhaustiveness of data collated, we highlight that InvaCost should be considered as the most current, standardised, accurate and globally representative repository of various economic losses and expenditures documented for the largest possible set of invaders. We are aware that our database can be improved in at least three ways.

First, InvaCost mostly does not include publications and reports not yet available in electronic format and/or using non-English language, leaving open the possibility of increasing data comprehensiveness and limiting potential biases. Indeed, local reports as well as research results from some countries (e.g., China, Russia) are likely to be published in non-English language^37^. Again, accessing grey literature is challenging as it is not systematically digitalised and/or included in well-curated bibliographic databases^29^. We strongly encourage future users of InvaCost to help gathering this currently unreachable information when possible. Furthermore, some mistakes might have occurred despite our best efforts when constructing InvaCost. In this regard, we advocate for regular public updates of InvaCost in order to improve it both quantitatively (by adding currently inaccessible or missed information) and qualitatively (if errors are identified).

Second, as the distribution and impacts of invaders are inherently dynamic for a number of reasons^38^, InvaCost should further consider the status of the species recorded for their economic impacts in order to improve both the relevance and the usefulness of the database. As an illustration, InvaCost likely includes invasive populations currently extirpated from particular areas after successful eradication campaign(s) as well as those still established but for which impacts are locally reduced as a result of management efforts. Attempting to obtain and integrate such information into InvaCost was beyond the scope of this work. Nonetheless, it should be reciprocally beneficial to establish connections between InvaCost and other databases such as the GRIIS that provides a harmonised, open source, multi-taxon database including verified information on the continued presence of introduced and invasive species for most countries^20^. In light of such additional information, the value of InvaCost will be its application for policy purposes, such as identification of exotic invaders that are currently associated with economic losses in particular areas. Also, crossing information between databases may allow the refinement of the descriptor ‘Spatial scale’ we propose here.

Third, we would recommend, for a future updated version of InvaCost that would require screening back all the materials, to improve the ‘Acquisition method’, ‘Implementation’ and ‘Reliability’ descriptors, to pay attention to the specificity of “avoided costs” and to create a new descriptor for ‘non-market values’. We detail these possibilities below.

#### Improving descriptors

An improved version of the ‘Acquisition method’ could lead to a subdivision of the *extrapolation* category into *spatial*, *temporal* and *spatio-temporal extrapolation*. This would allow simultaneous refinement from the currently binary ‘Implementation’ descriptor (*observed* vs *potential*) into several levels of certainty regarding the incurred cost (*e.g*. taking into consideration the temporality (past/current or predicted) of the onset of the cost and of the status of the invasive species in the study area). The next step for deeming the ‘Reliability’ of the cost estimates recorded in InvaCost would consist of assessing the repeatability of the methodology used, by adapting the approach previously developed by Bradshaw *et al*.^14^. The latter evidenced that assuming the reproducibility of published methods should not rely only on the nature of the materials and recognized the qualitative nature of the procedure, although applying this approach to InvaCost was constrained by the large sample size and high diversity in our database (Bradshaw *et al*.’s study focused on a single taxonomic class). Also, because InvaCost involves several collaborators and potential future contributors, consistent and objective criteria should be further defined to cope with the large array of materials, methods and situations encountered.

#### Avoided costs

Introducing certain actions against biological invasions leads to avoided costs. Such avoided costs are sometimes evaluated, for instance to examine the relevance of different potential actions or to assess the effectiveness of an action that was taken. However, avoided costs cover a great variety of situations and require a careful consideration for future analysis, even if they do not have to be analysed separately from the other economic costs gathered in InvaCost. For instance, in the case of hypothetical actions, avoided costs can be considered as minimum estimates of the “real” costs (if they are unknown). However, in the case of completed or planned actions, the reported data should be the original costs (if known) minus the avoided costs, because the latter do no longer exist. Some avoided costs are probably already included in InvaCost but they are likely underestimated because keywords such as “savings” or “benefits” were not included in the search strings. Also, even if they are sometimes mentioned as “benefits” in the literature, care should be taken not to confuse these avoided-costs with the benefits incurred by direct use or exploitation of invasive species. The latter have been ignored in InvaCost since they were relatively few (and beyond of the scope of this database), but might constitute a twin project.

#### A new ‘Non-market values’ descriptor

The means dedicated to preventing or managing an invasion (*e.g*. manual removal of invasive plants) and certain economic *losses and damage* due to an invasion (*e.g*. the value of crop losses or the repair costs of damaged infrastructures due to an invasive insect) are observable on markets. However, some costs are not observable on markets but can be translated in monetary terms using several valuation methods – for instance, the willingness to pay for the conservation of a native species that is impacted by an invasive species is considered as the value given by a group of people to preserving the native species (*i.e*. the value that would be lost if this native species was impacted). We recognize the importance of informing the public about “non-market values”, as giving an economic value to ecosystems or biodiversity can be a way of recognising and taking them into account in public decision-making processes^39^, but attention should be paid to the issues linked to their assessment^40,41^. Among others, the different methods for assessing non-market values do not necessarily capture the same aspects of the values, so the resulting estimates might be different. Moreover, the very principle of giving a value to “benefit from nature” through economic valuation is not necessarily acknowledged by the entirety of scientific and civil communities^39,42^. For future analysis, the ‘non-market values’ should not be systematically aggregated with the other economic costs gathered in InvaCost. It is to note that while some non-market values are probably already included in InvaCost within the *losses* and *damage* ‘Type of cost’, the loss of non-market values is probably largely underestimated in the database because they were not the primary focus of InvaCost and therefore the related keywords were not included in the search strings.

These possible ways of improvement call for completion and/or refinement of existing entries as well as integration of newly published or acquired data by future contributors in InvaCost, with the aim to consolidate its long-term relevance (cf. Usage Note paragraph).

## Data Records

All collected and examined references along with their bibliographic details and/or links for on-line access are recorded in an Excel workbook called *InvaCost_references* uploaded on Figshare^30^. This workbook consists of nine spreadsheets so that each bibliographic source considered (namely WoS, Google Scholar (GS), Google search engine (Go), and Targeted collection (TC)) is represented by two spreadsheets. The first four spreadsheets contain the complete list of potentially relevant materials obtained after applying specific search strings (*WoS-collected_refs*, *GS-collected_refs*, *Go-collected_refs*) or targeted searches (*TC-collected_refs*). The next four spreadsheets (*WoS-selected_refs*, *GS-selected_refs*, *Go-selected_refs*, and *TC-selected_refs*) comprise the list of relevant materials selected for final full-text screening. When a relevant material was found in more than one bibliographic repository, we considered it once only, as coming only from the first repository where it was chronologically recorded (respectively *WoS*, *GS*, *Go* and *TC*). The last spreadsheet (*Cited references*) contains the cited references collected during the full-text analysis of relevant materials. In each spreadsheet, each reference recorded is associated with a ‘Reference_ID’ that allows correspondence with InvaCost^30^. Cells with missing or unavailable information were marked as ‘NA’.

All cost estimates gathered in the abovementioned references were compiled in a second Excel workbook called *InvaCost_database* uploaded on Figshare^30^. It contains a dataset with a spreadsheet recording all the cost estimates arranged in such a way that each line refers to information retrieved from one bibliographic reference and related to a single cost estimate associated with one taxonomic group (generally at specific level) in one determined location (regardless of the geographic scale), and related to one specific period of time. Each entry is associated with a host of information provided for detailed characterisation of each cost recorded (Online-only Table 1, Table 2). Missing information was marked as ‘NA’. A second spreadsheet contains all the information used to standardise the cost estimates to 2017US$.

## Technical Validation

Considerable effort was made to ensure the highest possible degree of reliability in our database development. Each step was undertaken by at least two researchers to mitigate individual subjectivity when dealing with the documents. The whole process carefully followed the recommendations on rule-based search operations for collecting and synthesising relevant evidence^43^, as those reported in PRISMA (Preferred Reporting Items for Systematic Reviews and Meta-Analyses)^44^. Furthermore, extensive literature searches within several major bibliographic resources ensured that the collected sample was representative to the greatest extent possible. We also accounted for possible publication bias (*i.e*., the propensity for journals to publish studies with positive, hypothesis-affirming, or significant results rather than negative, contentious, or non-significant findings^45^) by searching both published and grey literature.

We also circumvented inherent limitations that appear when working with web search engines (e.g., the threshold for repetitive activity that triggers an automated block to a user’s IP address) by using IP-mirroring software. We also made sure when systematically accessing online systems that their terms of use were not violated. Furthermore, we followed the recommendation made by Haddaway *et al*.^32^ to check all viewable search results from all search engines used (contrary to the common practice of considering only the first 50–100 search results), leading to an improvement in both the transparency and coverage of the search process, especially with respect to grey literature. To validate our reference selection procedures, we ensured that no potentially relevant material would remain in the host of collected references that were considered as not relevant. For this purpose, we checked five random sub-samples (each sub-sample comprised at least 100 documents) of the list of non-relevant references to ensure that no potentially relevant material was excluded. As previously mentioned, each step of the entire process was systematically double-checked by at least two colleagues

## Usage Notes

InvaCost is expected to be of interest for a broad range of actors directly or indirectly interested in biological invasions (governments, researchers, conservation agencies, etc.). We stored the full dataset in a public repository to facilitate global access. We wish to highlight that InvaCost is open to corrections and updates from authors as well as any interested contributor through a systematic, standardised process. We are willing to receive any feedback that could improve our database. Any reader or user can therefore add new information and/or correct existing ones within InvaCost. The contributors are expected to send either a message, or data as a spreadsheet containing specific information corresponding to each column (Online-only Table 1) of InvaCost to our e-mail address (updates@invacost.fr). We encourage the future contributors to give as many narrative elements as possible in the ‘Details’ column that can contribute to better understanding of the cost estimates or to support choices made for completing the database, in order to allow backtracking investigations and facilitate the review process.

Contributors should provide access to the document from which information was taken. We strive to establish a collaborative community (including experts as well as non-expert contributors) and an online platform in order to sustain regular reviews of InvaCost. The intent is to ensure transparency of the process as well as relevance of this database as it is expanded and updated. Regularly-updated versions will be dated and permanently stored in the same repository as the original version, with a unique stable DOI and unique version numbering for each release. Each contributor bringing relevant additional information will be acknowledged in the updated version that will be released in the online repository (Column ‘Contributors’). Contributors are also encouraged to contact the authors in order to obtain supplementary information on how to use or update the database in case that information is not available here.

Users interested in working on the data described here are also asked to cite this manuscript as well as the specific version release of the database used, along with its DOI if necessary. The latter information should be systematically provided with the updated version downloaded from the public repository. We emphasise that users who aim to perform statistical analyses should take care to extract (from the database) and prepare relevant information based on the research question. One should keep in mind that duplicate or overlapping cost entries (*e.g*. multiple cost estimates for a single invasive species in a same location over a similar period) may exist in the database, and that these should be identified before any analysis. Importantly, neither the raw cost estimates (‘Raw_cost_estimate_2017_USD_exchange_rate’, ‘Raw_cost_estimate_2017_USD_PPP’) nor the cost estimates per years (‘Cost_estimate_per_year_2017_USD_exchange_rate’, ‘Cost_estimate_per_year_2017_USD_PPP’) should be simply summed since they do not necessarily cover the same time range or spatial scale, and they were not necessarily categorised within a working framework directly implementable for all types of studies. These columns should be used with complementary information on the ‘Period of estimation’, ‘Time_range’ and ‘Occurrence’ columns to take into account the temporal extent of the costs. Additionally, the *diverse*/*unspecified* nature of information retrieved (see previous sections) requires filtering out the data at hand before robustly quantifying the respective part of each descriptive category in the total cost. Furthermore, the spatial scale considered in InvaCost provides only an order of magnitude of the geographic extent considered for each cost estimate. Further exploring the economic costs of invasions based on data compiled in InvaCost should therefore ideally integrate specific information on the area dimensions actually impacted by the invasive species.

## Supplementary information

Supplementary Information 1

Supplementary Information 2

Supplementary Information 3

## Data Availability

The R script used in this manuscript to deal with the references collected from the WoS is provided as additional information (Supplementary file 3).

## Online-only Table

**Online-only Table 1:** Description of each type of information (by alphabetical order) provided for each cost estimate recorded in InvaCost. The categories used for each descriptive variable are italicised. Except for ‘Taxonomy’ that groups several columns, each row corresponds to a specific column in the database.

Column title	Description
Acquisition method	The method used to provide the cost estimate as a *report/estimation* (gathered or derived from field-based information) or *extrapolation* (cost modelled beyond the original spatial and/or temporal observation range)
Applicable year	The year of the ‘Currency’ value (not the year of the cost occurrence) considered for the conversion/standardization of the cost estimate
Authors	The authors of the material analysed
Availability	The accessibility of the material as a searchable document (*yes*/*no*)
Benefit value(s)	The mention of if any benefit value was found in the analysed material (*yes/no*); the figure was not recorded or described as being out of the scope of InvaCost
Common name	The non-scientific (or vernacular) name(s) provided by the authors, or by the International Union for Conservation of Nature (IUCN) when not provided
Contributors	The name of collaborator(s) having recorded the cost estimate; currently, it is only the initials of the authors, but each future contributor will be consistently acknowledged here
Cost estimate per year local currency	The ‘Raw cost estimate local currency’ transformed to a cost estimate per year of the ‘Period of estimation’ (obtained by dividing the raw cost estimate by the number of years* of the ‘Period of estimation’)
Cost estimate per year 2017 USD exchange rate	The ‘Cost estimate per year local currency’ standardised from local ‘Currency’ and ‘Applicable year’ to 2017 USD based on ER** (See the formula in the ‘Standardisation of cost data’ section)
Cost estimate per year 2017 USD PPP	The ‘Cost estimate per year local currency’ standardised from local ‘Currency’ and ‘Applicable year’ to 2017 USD based on PPP*** (See the formula in the ‘Standardisation of cost data’ section)
Cost ID	A unique numerical identifier for the cost estimate
Currency	The currency of the ‘Raw cost estimate local currency’ as extracted from the material
Details	When necessary, narrative elements deemed important either to understand the cost estimate or to support choices made for completing the database; this column was left unchanged from the original entries in order to allow trace-back investigations
Environment	The type of habitat (*aquatic*, *terrestrial*, *semi-aquatic*) where the cost estimate occurred
Geographic region	The geographical region(s) where the cost estimate occurred (*Africa*, *Asia*, *Central America*, *Europe*, *North America*, *Oceania-Pacific islands*, *South America*)
Impacted sector	The sector impacted by the cost estimate in our socio-ecosystems (e.g. *agriculture*, *health*, *public and social welfare*; see Table 3 for details on each category)
Implementation	This states – at the time of the estimation – whether the reported cost was actually *observed* (i.e. cost actually incurred) or *potential* (i.e. not incurred but expected cost)
Location	When provided, the precise location (e.g., region, city, area) where the cost estimate occurred
Max Raw cost estimate local currency	The higher boundary of the ‘Raw cost estimate local currency’ (if a range of estimates was provided by the authors)
Method reliability	The assessment of the methodological approach used for cost estimation as of *(i) high* reliability if either provided by officially pre-assessed materials (peer-reviewed articles and official reports) or the estimation method was documented, repeatable and/or traceable if provided by other grey materials, or *(ii) low* reliability if not (see Fig. 2 for more details)
Min Raw cost estimate local currency	The lower boundary of the ‘Raw cost estimate local currency’ (if a range of estimates was provided by the authors)
Occurrence	The status of the cost estimate as *potentially ongoing* (if the cost can be expected to continue over time) or *one-time* (if the cost was explicitly considered as over by the authors)
Official country	The country or official territory where the cost is incurred; sometimes, this is not congruent with the geographic regions as some territories (e.g. Overseas areas) are situated on other continents than their country of attachment
Period of estimation	If provided, the exact period of time covered by the costs estimated, otherwise the raw formulation (e.g. late 90’s, during 5 years)
Previous materials	If any, the list of successive materials checked before reaching the material originally providing the cost estimate
Probable starting year	The year range in which the cost is known or assumed to occur. When not explicitly provided by the authors, we mentioned ‘unspecified’ in both columns unless the authors provided a clear duration time. In this case, we considered the ‘Publication year’ as a reference for the probable starting/ending year from which we added/subtracted the number of years* of the ‘Period of estimation’. In the case of a cost estimate provided for a one-year period straddling two calendar years, we mentioned the latest year of the cost occurrence in both columns. When vague formulations were used (e.g. early 90’s), we still translated them in probable ending/starting year (e.g. 1990–1995). We will harmonise the way these specific cases are dealt with when reviewing and validating new lines proposed by new contributors.
Probable ending year	
Publication year	The publication year of the material analysed
Raw cost estimate local currency	The cost estimate directly retrieved from the analysed materials
Raw cost estimate 2017 USD exchange rate	The ‘Raw cost estimate local currency’ standardised from local ‘Currency’ and ‘Applicable year’ to 2017 US$ based on ER** See the formula in the Standardisation of cost data section
Raw cost estimate 2017 USD PPP	The ‘Raw cost estimate local currency’ standardised to 2017 US$ based on PPP*** See the formula in the Standardisation of cost data section
Reference ID	The numerical identifier of the material analysed, which - along with ‘Repository’ - allows correspondence with the *InvaCost_references* file^30^ that provides bibliographic details
Reference title	The title of the material analysed
Repository	The original source of each material: ‘Web of Science (*WoS*)’, ‘Google Scholar (*GS*)’, ‘Google search engine (*Go*)’ or ‘Targeted collection (*TC*)’
Spatial scale	The spatial scale considered for estimating the cost: global (worldwide-scale), intercontinental (sites from two or more geographic regions) continental (‘geographic region’ level), regional (several countries within a single geographic region), country, site (for cost evaluated at intra-country level, including USA states) and unit (for costs evaluated for a well-defined surface area or entity)
Taxonomy	For each species recorded, the taxonomic information from kingdom to species level using the Global Biodiversity Information Facility (GBIF) as a reference
Time range	The time range considered by the authors for providing the cost estimate: *year* (when costs were if the estimate is given yearly or for a period up to one year) or *period* (when costs were provided for a period exceeding a year)
Type of cost	The type of the cost includes: damages and losses incurred by an invasion (*e.g*. damage repair, medical care, value of crop losses), or means dedicated to understand or predict (*e.g*. research), prevent (*e.g*. education, biosecurity), early detect (*e.g*. monitoring, surveillance) and/or manage (*e.g*. control, eradication) the invaders
Type of applicable year	The assessment of the applicable year as *effective* if explicitly stated by the authors or *publication year* if no explicit information provided
Type of material	The type of material analysed (*i.e*. scientific *peer-reviewed article* or grey literature); for grey literature, the exact nature of the material was indicated (*e.g*., *official report*, *press release*)

*The number of years of the ‘Period of estimation’ is the difference between the ‘Probable ending year’ and the ‘Probable starting year”; when we had no complete or exact information on the period of costs, we considered the publication year as a starting and/or ending year, and approximated the duration time based on either the available information provided by the authors or a one-year duration when no information was available)

**Market exchange rate (local currency unit per US$) provided by the World Bank Open Data (available at https://data.worldbank.org/indicator/PA.NUS.FCRF?end=2017&start=1960).

***Purchase Power Parity (local currency unit per US$) provided by the World Bank Open Data (available at https://data.worldbank.org/indicator/PA.NUS.PPP?end=2017&start=1990) and the Organisation for Economic Cooperation and Development (available at https://data.oecd.org/conversion/purchasing-power-parities-ppp.htm).
