# Supplementary material for: InvaCost, a public database of the economic costs of biological invasions worldwide
Source: Sci Data. 2020 Sep 8;7:277. doi: 10.1038/s41597-020-00586-z (PMC7479195; doi:10.1038/s41597-020-00586-z)
Supplement: Supplementary file 1 — Supplementary Information 1 [file 41597_2020_586_MOESM1_ESM.pdf]

Supplementary file 1. Search strings implemented in the ISI Web of Science (A) and PubMed repository (B) to reach costs associated to invasive mosquito species as part of the targeted collection. The whole procedure carefully followed the recommendations from PRISMA (Preferred Reporting Items for Systematic Reviews and Meta-Analyses) statements (C).

(A) Search string used in the ISI Web of Science platform (https://webofknowledge.com/):

("cost effectiveness" OR "cost-effectiveness" OR monetary OR dollars OR euros OR sterling OR DALY OR expenditur* OR economi* OR "cost of illness" OR "cost-of-illness") AND (TS=(zika OR chikungunya OR dengue OR "yellow fever" OR albopictus OR aegypti))

(B) Search string used in the PubMed repository (https://www.ncbi.nlm.nih.gov/pubmed/)

cost-effectiveness[Title/Abstract] OR cost effectiveness[Title/Abstract] OR monetary[Title/Abstract] OR dollars[Title/Abstract] OR euros[Title/Abstract] OR sterling[Title/Abstract] OR DALY[Title/Abstract] OR expenditur*[Title/Abstract] OR economi*[Title/Abstract] OR cost of illness[Title/Abstract] OR cost-of-illness[Title/Abstract]) AND (**zika[Title/Abstract] OR chikungunya[Title/Abstract] OR dengue[Title/Abstract] OR yellow fever[Title/Abstract] OR albopictus[Title/Abstract] OR aegypti[Title/Abstract])**

(C)

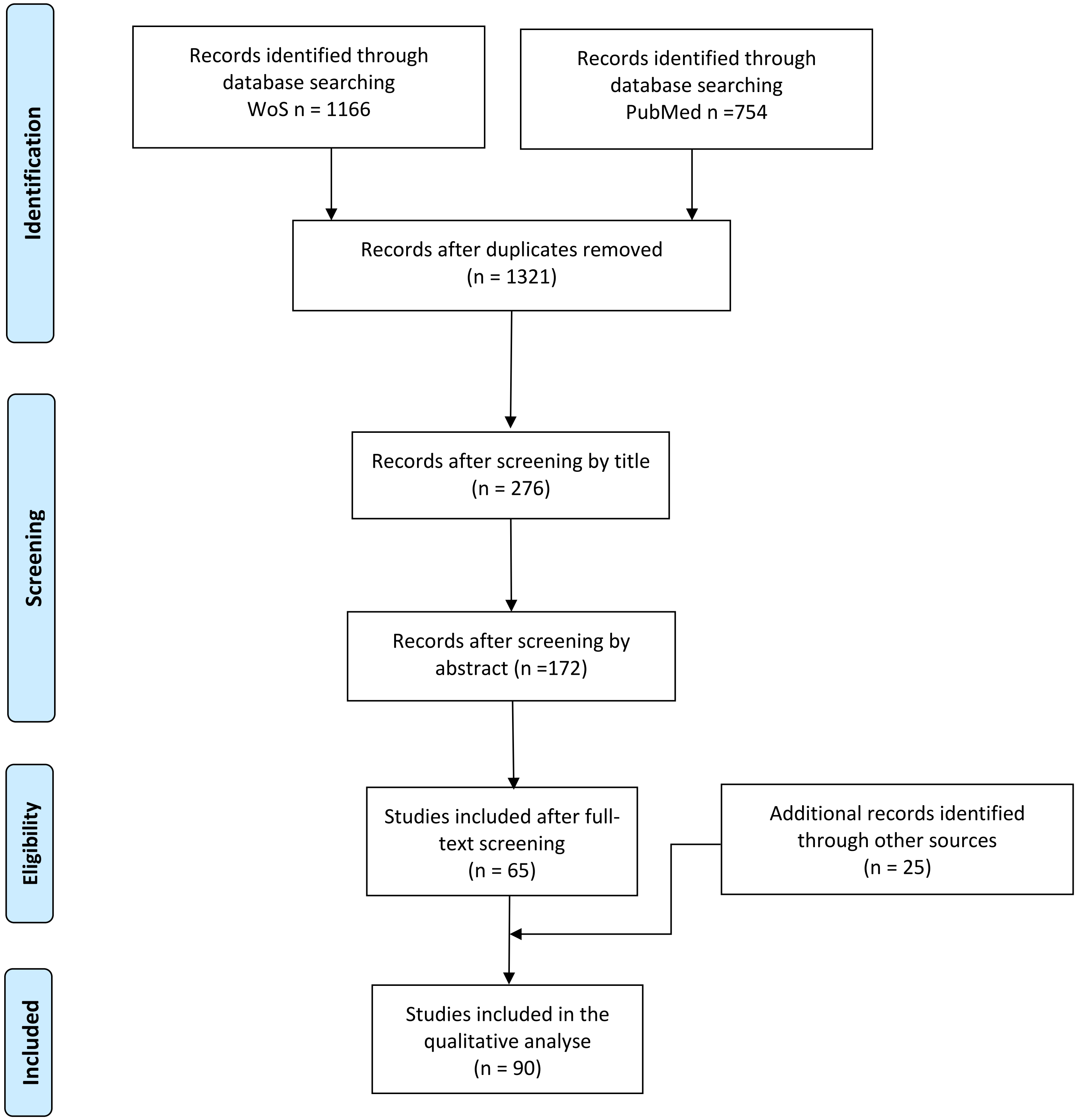
Identification
Screening
Eligibility
Included
Records identified through database searching WoS n = 1166
Records identified through database searching PubMed n =754
Records after duplicates removed (n = 1321)
Records after screening by title (n = 276)
Records after screening by abstract (n =172)
Studies included after full-text screening (n = 65)
Additional records identified through other sources (n = 25)
Studies included in the qualitative analyse (n = 90)
