# Supplementary material for: InvaCost, a public database of the economic costs of biological invasions worldwide
Source: Sci Data. 2020 Sep 8;7:277. doi: 10.1038/s41597-020-00586-z (PMC7479195; doi:10.1038/s41597-020-00586-z)
Supplement: Supplementary file 2 — Supplementary Information 2 [file 41597_2020_586_MOESM2_ESM.pdf]

Supplementary file 2

Distribution of the cost data recorded in INVACOST according to the 'Geographic region' of the cost estimates. *Nref*: Number of materials (in grey); *Ncost*: Number of cost estimates (in blue); *Nhigh*: Number of high reliability estimates (in green); *Ntaxa*: Number of taxonomic units (in red) that refers either to a single species or, if any, unique group of species for which specific contribution to the whole cost is not possible. 'Mixed' contains data concomitantly associated with two or more geographic regions.

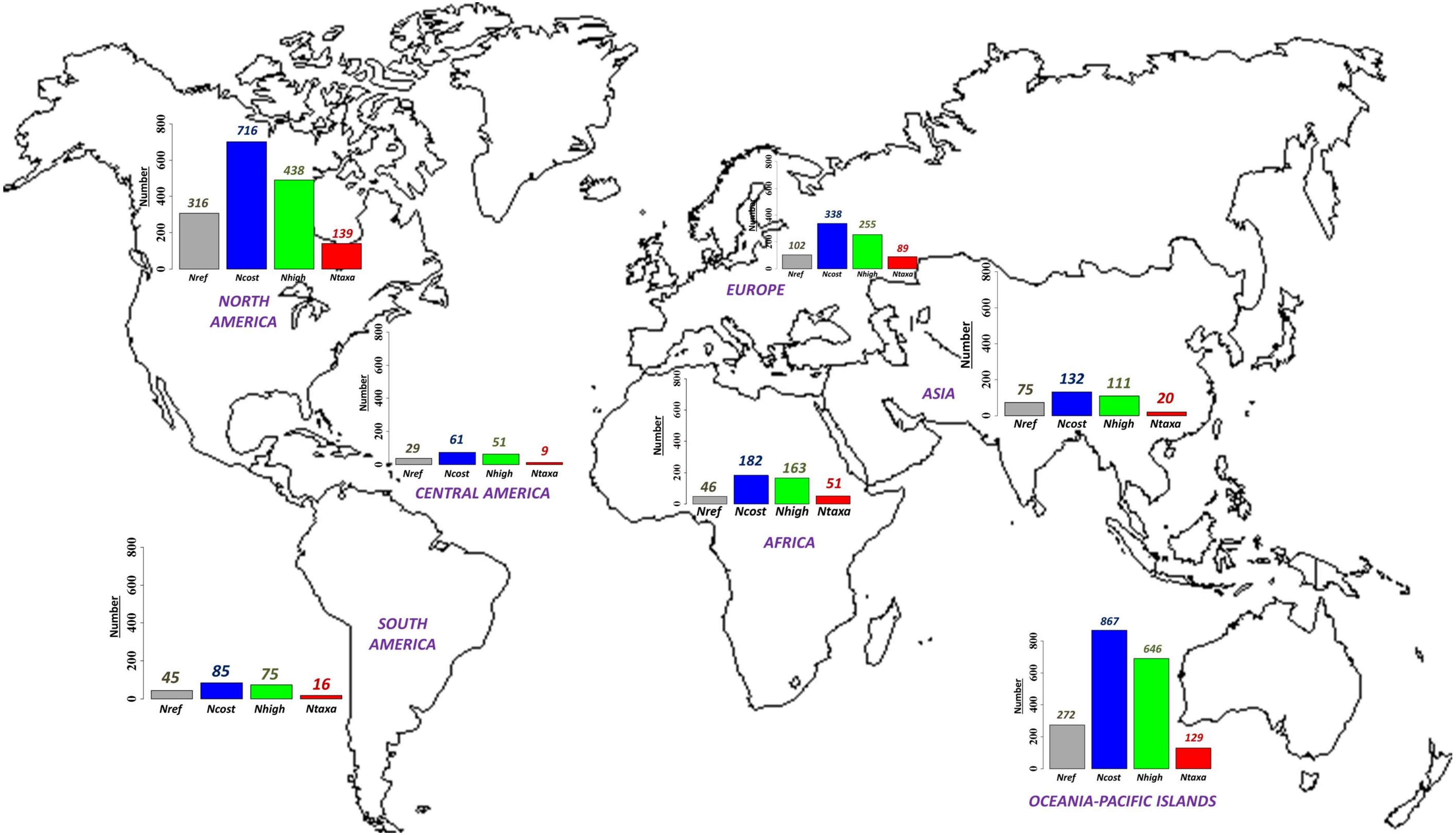
NORTH AMERICA
Number
0
200
400
600
800
316
716
438
139
Nref
Ncost
Nhigh
Ntaxa
EUROPE
102
338
255
89
Nref
Ncost
Nhigh
Ntaxa
ASIA
Number
75
132
111
20
Nref
Ncost
Nhigh
Ntaxa
CENTRAL AMERICA
Number
29
61
51
9
Nref
Ncost
Nhigh
Ntaxa
AFRICA
Number
46
182
163
51
Nref
Ncost
Nhigh
Ntaxa
SOUTH AMERICA
Number
45
85
75
16
Nref
Ncost
Nhigh
Ntaxa
OCEANIA-PACIFIC ISLANDS
Number
272
867
646
129
Nref
Ncost
Nhigh
Ntaxa
