# Supplementary material for: InvaCost, a public database of the economic costs of biological invasions worldwide
Source: Sci Data. 2020 Sep 8;7:277. doi: 10.1038/s41597-020-00586-z (PMC7479195; doi:10.1038/s41597-020-00586-z)
Supplement: Supplementary file 3 — Supplementary Information 3 [file 41597_2020_586_MOESM3_ESM.pdf]

```
###bibl <- dataset (.csv file) with all information extracted from the topic (title = column 2, abstract = column 3, keywords = column 4, keywords plus = column 5)

###upload dataset "InvaCost_Refs" with all information extracted from the topic (title = column 2, abstract = column 3, keywords = column 4, keywords plus = column = 5
bibl <- read.csv2("InvaCost_Refs.csv", header=TRUE)

### total number of references collected from WOS
publications <- 16875

##########searching words of interest in each component of the topic##########

###search in title
paperlist <- matrix(0,publications,2)
colnames(paperlist) <- as.character(c("econom_t" ,"invasi_t"))

for(i in c(1:publications)) {

	title <- unlist(as.character(bibl[i,2]))

if (!length(grep("econom", title, ignore.case=TRUE, value=TRUE))==0) {paperlist[i,"econom_t"] <-1}
if (!length(grep("cost", title, ignore.case=TRUE, value=TRUE))==0) {paperlist[i,"econom_t"] <-1}
if (!length(grep("monetary", title, ignore.case=TRUE, value=TRUE))==0) {paperlist[i,"econom_t"] <-1}
if (!length(grep("\\$", title, ignore.case=TRUE, value=TRUE))==0) {paperlist[i,"econom_t"] <-1}
if (!length(grep("\u20AC", title, ignore.case=TRUE, value=TRUE))==0) {paperlist[i,"econom_t"] <- 1}
if (!length(grep("\\£", title, ignore.case=TRUE, value=TRUE))==0) {paperlist[i,"econom_t"] <-1}
if (!length(grep("dollar", title, ignore.case=TRUE, value=TRUE))==0) {paperlist[i,"econom_t"] <-1}
if (!length(grep("euro", title, ignore.case=TRUE, value=TRUE))==0) {paperlist[i,"econom_t"] <-1}
if (!length(grep("pound", title, ignore.case=TRUE, value=TRUE))==0) {paperlist[i,"econom_t"] <-1}
if (!length(grep("invasi", title, ignore.case=TRUE, value=TRUE))==0) {paperlist[i,"invasi_t"] <- 1}
if (!length(grep("alien", title, ignore.case=TRUE, value=TRUE))==0) {paperlist[i,"invasi_t"] <- 1}
if (!length(grep("non-indigenous", title, ignore.case=TRUE, value=TRUE))==0) {paperlist[i,"invasi_t"] <- 1}
if (!length(grep("non-native", title, ignore.case=TRUE, value=TRUE))==0) {paperlist[i,"invasi_t"] <- 1}
if (!length(grep("exotic", title, ignore.case=TRUE, value=TRUE))==0) {paperlist[i,"invasi_t"] <- 1}
if (!length(grep("introduced", title, ignore.case=TRUE, value=TRUE))==0) {paperlist[i,"invasi_t"] <- 1}
if (!length(grep("naturali", title, ignore.case=TRUE, value=TRUE))==0) {paperlist[i,"invasi_t"] <- 1}
if (!length(grep("invader", title, ignore.case=TRUE, value=TRUE))==0) {paperlist[i,"invasi_t"] <- 1}

}

write.csv (paperlist, file=("results_title.csv"))
```

```
###search in abstract
paperlist <- matrix(0,publications,2)
colnames(paperlist) <- as.character(c("econom_a" ,"invasi_a"))

for(i in c(1:publications)) {
	abstract <- unlist(as.character(bibl[i,3]))

if (!length(grep("econom", abstract, ignore.case=TRUE, value=TRUE))==0) {paperlist[i,"econom_a"] <-
paperlist[i,"econom_a"] + 1}
if (!length(grep("cost", abstract, ignore.case=TRUE, value=TRUE))==0) {paperlist[i,"econom_a"] <-
paperlist[i,"econom_a"] + 1}
if (!length(grep("monetary", abstract, ignore.case=TRUE, value=TRUE))==0) {paperlist[i,"econom_a"]
<-paperlist[i,"econom_a"] + 1}
if (!length(grep("\\$", abstract, ignore.case=TRUE, value=TRUE))==0) {paperlist[i,"econom_a"] <-
paperlist[i,"econom_a"] + 1}
if (!length(grep("\u20AC", abstract, ignore.case=TRUE, value=TRUE))==0) {paperlist[i,"econom_a"] <-
paperlist[i,"econom_a"] + 1}
if (!length(grep("\\£", abstract, ignore.case=TRUE, value=TRUE))==0) {paperlist[i,"econom_a"] <-
paperlist[i,"econom_a"] + 1}
if (!length(grep("dollar", abstract, ignore.case=TRUE, value=TRUE))==0) {paperlist[i,"econom_a"] <-
paperlist[i,"econom_a"] + 1}
if (!length(grep("euro", abstract, ignore.case=TRUE, value=TRUE))==0) {paperlist[i,"econom_a"] <-
paperlist[i,"econom_a"] + 1}
if (!length(grep("pound", abstract, ignore.case=TRUE, value=TRUE))==0) {paperlist[i,"econom_a"] <-
paperlist[i,"econom_a"] + 1}
if (!length(grep("invasi", abstract, ignore.case=TRUE, value=TRUE))==0) {paperlist[i,"invasi_a"] <-
paperlist[i,"invasi_a"] + 1}
if (!length(grep("alien", abstract, ignore.case=TRUE, value=TRUE))==0) {paperlist[i,"invasi_a"] <-
paperlist[i,"invasi_a"] + 1}
if (!length(grep("non-indigenous", abstract, ignore.case=TRUE, value=TRUE))==0)
{paperlist[i,"invasi_a"] <- paperlist[i,"invasi_a"] + 1}
if (!length(grep("non-native", abstract, ignore.case=TRUE, value=TRUE))==0) {paperlist[i,"invasi_a"] <-
paperlist[i,"invasi_a"] + 1}
if (!length(grep("exotic", abstract, ignore.case=TRUE, value=TRUE))==0) {paperlist[i,"invasi_a"] <-
paperlist[i,"invasi_a"] + 1}
if (!length(grep("introduced", abstract, ignore.case=TRUE, value=TRUE))==0) {paperlist[i,"invasi_a"]
<- paperlist[i,"invasi_a"] + 1}
if (!length(grep("naturali", abstract, ignore.case=TRUE, value=TRUE))==0) {paperlist[i,"invasi_a"] <-
paperlist[i,"invasi_a"] + 1}
if (!length(grep("invader", abstract, ignore.case=TRUE, value=TRUE))==0) {paperlist[i,"invasi_a"] <-
paperlist[i,"invasi_a"] + 1}

}

write.csv (paperlist, file=("results_abstract.csv"))
```

```
###search in keywords
paperlist <- matrix(0,publications,2)
colnames(paperlist) <- as.character(c("econom_k" ,"invasi_k"))

for(i in c(1:publications)) {
	keywords <- unlist(as.character(bibl[i,4]))

if (!length(grep("econom", keywords, ignore.case=TRUE, value=TRUE))==0) {paperlist[i,"econom_k"]
<-paperlist[i,"econom_k"] + 1}
if (!length(grep("cost", keywords, ignore.case=TRUE, value=TRUE))==0) {paperlist[i,"econom_k"] <-
paperlist[i,"econom_k"] + 1}
if (!length(grep("monetary", keywords, ignore.case=TRUE, value=TRUE))==0)
{paperlist[i,"econom_k"] <-paperlist[i,"econom_k"] + 1}
if (!length(grep("\\$", keywords, ignore.case=TRUE, value=TRUE))==0) {paperlist[i,"econom_k"] <-
paperlist[i,"econom_k"] + 1}
if (!length(grep("\u20AC", keywords, ignore.case=TRUE, value=TRUE))==0) {paperlist[i,"econom_k"]
<- paperlist[i,"econom_k"] + 1}
if (!length(grep("\\£", keywords, ignore.case=TRUE, value=TRUE))==0) {paperlist[i,"econom_k"] <-
paperlist[i,"econom_k"] + 1}
if (!length(grep("dollar", keywords, ignore.case=TRUE, value=TRUE))==0) {paperlist[i,"econom_k"] <-
paperlist[i,"econom_k"] + 1}
if (!length(grep("euro", keywords, ignore.case=TRUE, value=TRUE))==0) {paperlist[i,"econom_k"] <-
paperlist[i,"econom_k"] + 1}
if (!length(grep("pound", keywords, ignore.case=TRUE, value=TRUE))==0) {paperlist[i,"econom_k"] <-
paperlist[i,"econom_k"] + 1}
if (!length(grep("invasi", keywords, ignore.case=TRUE, value=TRUE))==0) {paperlist[i,"invasi_k"] <-
paperlist[i,"invasi_k"] + 1}
if (!length(grep("alien", keywords, ignore.case=TRUE, value=TRUE))==0) {paperlist[i,"invasi_k"] <-
paperlist[i,"invasi_k"] + 1}
if (!length(grep("non-indigenous", keywords, ignore.case=TRUE, value=TRUE))==0)
{paperlist[i,"invasi_k"] <- paperlist[i,"invasi_k"] + 1}
if (!length(grep("non-native", keywords, ignore.case=TRUE, value=TRUE))==0) {paperlist[i,"invasi_k"]
<- paperlist[i,"invasi_k"] + 1}
if (!length(grep("exotic", keywords, ignore.case=TRUE, value=TRUE))==0) {paperlist[i,"invasi_k"] <-
paperlist[i,"invasi_k"] + 1}
if (!length(grep("introduced", keywords, ignore.case=TRUE, value=TRUE))==0) {paperlist[i,"invasi_k"]
<- paperlist[i,"invasi_k"] + 1}
if (!length(grep("naturali", keywords, ignore.case=TRUE, value=TRUE))==0) {paperlist[i,"invasi_k"] <-
paperlist[i,"invasi_k"] + 1}
if (!length(grep("invader", keywords, ignore.case=TRUE, value=TRUE))==0) {paperlist[i,"invasi_k"] <-
paperlist[i,"invasi_k"] + 1}

}

write.csv (paperlist, file=("results_keywords.csv"))
```

```
###search in keywords pluspaperlist <- matrix(0,publications,2)
colnames(paperlist) <- as.character(c("econom_kp" ,"invasi_kp"))

for(i in c(1:publications)) {
  keywordsPlus <- unlist(as.character(bibl[i,5]))

if (!length(grep("econom", keywordsPlus, ignore.case=TRUE, value=TRUE))==0)
{paperlist[i,"econom_kp"] <-paperlist[i,"econom_kp"] + 1}
if (!length(grep("cost", keywordsPlus, ignore.case=TRUE, value=TRUE))==0) {paperlist[i,"econom_kp"]
<-paperlist[i,"econom_kp"] + 1}
if (!length(grep("monetary", keywordsPlus, ignore.case=TRUE, value=TRUE))==0)
{paperlist[i,"econom_kp"] <-paperlist[i,"econom_kp"] + 1}
if (!length(grep("\\$", keywordsPlus, ignore.case=TRUE, value=TRUE))==0) {paperlist[i,"econom_kp"]
<-paperlist[i,"econom_kp"] + 1}
if (!length(grep("\u20AC", keywordsPlus, ignore.case=TRUE, value=TRUE))==0)
{paperlist[i,"econom_kp"] <- paperlist[i,"econom_kp"] + 1}
if (!length(grep("\\£", keywordsPlus, ignore.case=TRUE, value=TRUE))==0) {paperlist[i,"econom_kp"]
<-paperlist[i,"econom_kp"] + 1}
if (!length(grep("dollar", keywordsPlus, ignore.case=TRUE, value=TRUE))==0)
{paperlist[i,"econom_kp"] <-paperlist[i,"econom_kp"] + 1}
if (!length(grep("euro", keywordsPlus, ignore.case=TRUE, value=TRUE))==0)
{paperlist[i,"econom_kp"] <-paperlist[i,"econom_kp"] + 1}
if (!length(grep("pound", keywordsPlus, ignore.case=TRUE, value=TRUE))==0)
{paperlist[i,"econom_kp"] <-paperlist[i,"econom_kp"] + 1}
if (!length(grep("invasi", keywordsPlus, ignore.case=TRUE, value=TRUE))==0) {paperlist[i,"invasi_kp"]
<- paperlist[i,"invasi_kp"] + 1}
if (!length(grep("alien", keywordsPlus, ignore.case=TRUE, value=TRUE))==0) {paperlist[i,"invasi_kp"]
<- paperlist[i,"invasi_kp"] + 1}
if (!length(grep("non-indigenous", keywordsPlus, ignore.case=TRUE, value=TRUE))==0)
{paperlist[i,"invasi_kp"] <- paperlist[i,"invasi_kp"] + 1}
if (!length(grep("non-native", keywordsPlus, ignore.case=TRUE, value=TRUE))==0)
{paperlist[i,"invasi_kp"] <- paperlist[i,"invasi_kp"] + 1}
if (!length(grep("exotic", keywordsPlus, ignore.case=TRUE, value=TRUE))==0) {paperlist[i,"invasi_kp"]
<- paperlist[i,"invasi_kp"] + 1}
if (!length(grep("introduced", keywordsPlus, ignore.case=TRUE, value=TRUE))==0)
{paperlist[i,"invasi_kp"] <- paperlist[i,"invasi_kp"] + 1}
if (!length(grep("naturali", keywordsPlus, ignore.case=TRUE, value=TRUE))==0)
{paperlist[i,"invasi_kp"] <- paperlist[i,"invasi_kp"] + 1}
if (!length(grep("invader", keywordsPlus, ignore.case=TRUE, value=TRUE))==0)
{paperlist[i,"invasi_kp"] <- paperlist[i,"invasi_kp"] + 1}

}

write.csv (paperlist, file=("results_keywordsPlus.csv"))
```
